# Identifying potential causal effects of age at menopause: a Mendelian randomization phenome-wide association study

**DOI:** 10.1007/s10654-022-00903-3

**Published:** 2022-09-03

**Authors:** Maria C. Magnus, Maria Carolina Borges, Abigail Fraser, Deborah A. Lawlor

**Affiliations:** 1 Centre for Fertility and Health, Norwegian Institute of Public Health, P.O. Box 222, 0213 Skøyen, Oslo, Norway; 2 MRC Integrative Epidemiology Unit at the University of Bristol, Bristol, UK; 3 Population Health Sciences,, Bristol Medical School, Bristol, UK; 4 NIHR Bristol Biomedical Research Centre at the University Hospitals Bristol NHS Foundation Trust, University of Bristol, Bristol, UK

**Keywords:** MR-pheWAS, Menopause, Mendelian randomization

## Abstract

Age at natural menopause (ANM) is associated with a range of health-related traits, including bone health, female reproductive cancers, and cardiometabolic health. Our objective was to conduct a Mendelian randomization phenome-wide association study (MR-pheWAS) of ANM. We conducted a hypothesis-free analysis of the genetic risk score (GRS) for ANM with 18,961 health-related traits among 181,279 women in UK Biobank. We also stratified the GRS according to the involvement of SNPs in DNA damage response. We sought to replicate our findings in independent cohorts. We conducted a negative control MR-pheWAS among men. Among women, we identified potential effects of ANM on 221 traits (1.17% of all traits) at a false discovery rate (*P *value ≤ 5.83 × 10^–4^), and 91 (0.48%) potential effects when using Bonferroni threshold (*P *value ≤ 2.64 × 10^–6^). Our findings included 55 traits directly related to ANM (e.g. hormone replacement therapy, gynaecological conditions and menstrual conditions), and liver function, kidney function, lung function, blood-cell composition, breast cancer and bone and cardiometabolic health. Replication analyses confirmed that younger ANM was associated with HbA1c (adjusted mean difference 0.003 mmol/mol; 95% CI 0.001, 0.006 per year decrease in ANM), breast cancer (adjusted OR 0.96; 95% CI 0.95, 0.98), and bone-mineral density (adjusted mean difference − 0.05; 95% CI − 0.07, − 0.03 for lumbar spine). In men, 30 traits were associated with the GRS at a false discovery rate (*P *value ≤ 5.49 × 10^–6^), and 11 potential effects when using Bonferroni threshold (*P *value ≤ 2.75 × 10^–6^). In conclusion, our results suggest that younger ANM has potential causal effects on a range of health-related traits.

**Supplementary Information:**

The online version contains supplementary material available at 10.1007/s10654-022-00903-3.

## Introduction

Menopause marks the end of women´s reproductive lifespan and cessation of the endogenous female sex-hormone production from the ovaries. Higher age at natural menopause (ANM) is associated with lower risk of cardiovascular disease [1] and osteoporosis [2, 3], and a higher risk of female reproductive cancers [4–6]. Some of these findings have been further substantiated by studies looking at the role of hormone replacement therapy in the risk of these disorders [7].

Evidence regarding the risk of chronic diseases according to ANM come mostly from traditional observational studies, given it is not feasible to conduct experimental studies to manipulate age at menopause. Mendelian randomization (MR) analysis attempts to mimic randomized controlled trials by using genetic variants as instruments for the exposure of interest [8]. Under the assumption that genetic variants are randomly allocated at conception, this analytical approach minimizes the risk of bias due to confounding. Previous studies have used MR to examine the relationship of ANM with breast cancer, ovarian cancer, colorectal cancer and lung function [5, 9–11]. These studies have confirmed potential effects of later ANM on greater risk of breast cancer, and a surprising potential effect between earlier ANM and decreased airflow obstruction.

To identify novel relationship of interest, a hypothesis-free approach can be useful. It is common for research to continue exploring the same associations/effects. For example, a PubMed search for primary research papers of ANM with cardiovascular diseases and breast cancer identified 307 and 517 publications (see strategy presented in online methods), whereas a similar search for depression identified 49 publications. Reductions in endogenous sex hormones have been hypothesised to associate with a range of health outcomes beyond cardiovascular diseases, bone health and reproductive cancers but for these there are fewer and smaller studies. Previous MR phenome-wide association studies of body-mass index (BMI), smoking and age at menarche have provided novel evidence of effects on outcomes not previously identified as being associated with these exposures [12–14].

The objective of this study was to systematically investigate causal effects of ANM on health-related traits, by conducting a MR phenome-wide association study (MR-pheWAS).

## Participations and methods

### UK biobank

**Fig. 1:**
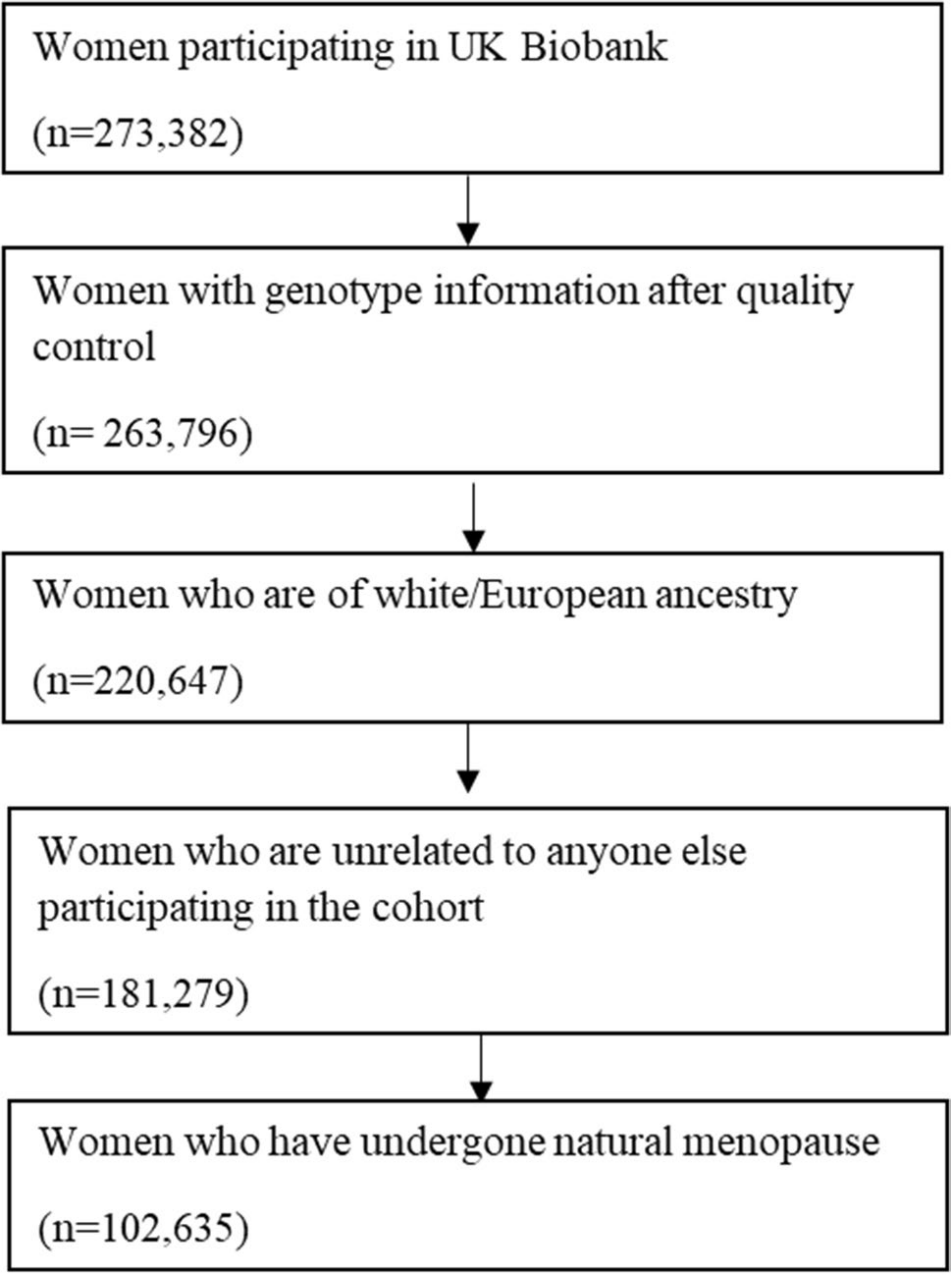
Study population

The MR-pheWAS was undertaken in the UK Biobank cohort. The UK Biobank cohort includes 503,325 people (273,453 women) between 40 and 69 years of age, who were recruited between 2006 and 2010, from 22 assessment centres across England, Scotland and Wales [15, 16]. The response rate was 5.5%, and all participants gave written informed consent. Participants were followed prospectively after enrolment using Hospital Episode Statistics data, as well as data from cancer registries and the Office of National Statistics. Genotyping was performed using the Affymetrix UK BiLEVE Axiom array on an initial 50,000 participants; the remaining 450,000 participants were genotyped using the Affymetrix UK Biobank Axiom^®^ array [17]. Quality control and imputation (to over 90 million SNPs, indels and large structural variants) was performed by the Wellcome Trust Centre for Human Genetics [17]. The current analysis included 181,279 unrelated genotyped women of European ancestry (Fig. 1). Relatedness was defined as 3rd degree relatives or closer [18].

### Identifying the genetic instruments for age at natural menopause

We identified genetic instruments for ANM from the most recent genome-wide association study (GWAS) [19]. This meta-analysis included 201,323 women of European ancestry, and identified 290 single-nucleotide polymorphisms (SNPs) that predicted ANM at the conventional GWAS threshold (*P *value < 5 × 10^−8^). We generated an externally weighted genetic risk score (GRS) as a weighted sum of the number of ANM decreasing alleles across 267 autosomal SNPs, weighted by the published GWAS effect estimates excluding UK Biobank. SNPs on the X chromosome were excluded to ensure comparability of the results for men and women. We conducted a sensitivity analysis restricting the GRS to SNPs with a minimum distance of 10,000 kb and a *R*^2^ < 0.001, which left us with 151 strictly unrelated SNPs. SNPs associated with ANM are highly enriched for genes in DNA damage response (DDR) pathways [9]. Therefore, we ran additional analyses with stratified GRS containing either DDR (104 SNPs) or non-DDR SNPs (163 SNPs) to explore whether MR-PheWAS findings were reflecting a direct effect of experiencing menopause or general cell aging.

### Phenome-wide Mendelian randomization analysis

We conducted a MR-pheWAS using the publicly available PHESANT software (version 0.17) which uses an automated rule-based method [20]. The decision rules start with identifying continuous, ordered categorical, unordered categorical or binary variable fields. After outcome pre-processing (continuous traits were inverse normal rank transformed to ensure they were normally distributed), PHESANT runs linear (continuous outcomes), logistic (binary outcomes), ordered logistic (ordered categorical outcomes), and multinomial logistic (unordered categorical outcomes) regression, with the weighted allele score for ANM as the exposure. Unordered categorical outcomes were analysed using multinomial logistic regression. All analyses are adjusted for age and the first 10 genetic principal components.

We used PHESANT to examine the association of the ANM GRS with 18,961 health-related traits. To identify potential causal effects of ANM, we used two approaches that account for the number of tests performed, to help us evaluate the strength of the evidence from our MR-pheWAS. First, we derived a *P *value threshold setting the false discovery rate at 5%. After ranking the results by *P *value, this threshold is calculated as *P*_t_(rank) = 0.05 × rank/*n*, where *n* is the total number of tests in the phenome scan and *rank* is the largest rank position with a *p *value less than *P*_t_. Second, we calculated a Bonferroni corrected *P *value threshold, by dividing 0.05 by the number of tests performed.

### Follow-up and replication analyses of MR-pheWAS findings

The first step in our follow-up of the MR-pheWAS findings was to estimate the magnitude of the causal effects for the outcomes we followed up for replication analysis, including liver function, measures of kidney function, low-density lipoprotein (LDL) cholesterol, HbA1c, forced vital capacity (FVC), percent body fat, type 2 diabetes, celiac disease, bone-mineral density and breast cancer. PHESANT measures the association of the GRS with the outcomes (estimates reflect the mean difference or log-odds in outcomes per unit increase in the GRS), one and two-sample MR analyses are required to estimate the magnitude of the causal effect of the exposure (per year decrease in ANM). We estimated the magnitude of potential causal effects of ANM in UK Biobank using one-sample MR. This required that we first estimated the genetically predicted ANM using linear regression, with ANM as the outcome and the GRS as the exposure. In the second step, we estimated the magnitude of the causal effect using linear or logistic regression analysis, with genetically predicted ANM as the exposure variable of interest.

We used published GWAS to replicate one finding in each of the main categories, including liver function (n = 61,089) [21], measures of kidney function (n = 133,413) [22], LDL cholesterol (n = 188,577) [23], HbA1c (n = 123,665) [24], bone-minderal density (n =  ~ 22,000) [25], breast cancer (122,977 cases and 105,974 controls) [26], FVC (n = 79,055) [27], percent body fat (n = 100,716) [28], type 2 diabetes (62,892 cases and 596,424 controls) [29] and coeliac disease (12,041 cases and 12,228 controls) [30]. Sex-specific GWAS results was only avaiable for bone-mineral density and breast cancer risk. The replication analyses were conducted using two-sample MR, with the main analyses using the inverse variance weighted method, which estimates effects by regession (linear or logistic) through summary results of the association of each SNP for ANM (selected in an identical way to the selection for the GRS) with ANM and with the outcome being explored, whilst forcing the regression line through zero [31]. We estimated the effect using MR-Egger (MR-Egger) regression, which does not force the intercept through zero. The estimate of the causal effect from the MR-Egger regression is unbiased if the strength of the gene-exposure association does not correlate with the strength of the bias due to horizontal pleiotropy (known as the Instrument Strength Independent of Direct Effect, or InSIDE assumption) [31]. A non-zero intercept from this regression model is an indicator of unbalanced horizontal pleiotropy [31]. Additional sensitivity analyses included simple and weighted mode-based and weighted median regression [32]. Concordance among estimates minimizes the risk of unbalanced pleiotropy.

### Negative control analysis

To further evaluate whether any findings were likely to reflect a direct effect of ANM and not general effects associated with biological aging that are shared between the sexes, we also ran the MR-pheWAS among men participating in UK Biobank (N = 155,709). We chose to do this negative control analysis because a large proportion of the SNPs included in the GRS for ANM among women were located in DDR genes, which may also reflect various aspects related to biological aging. Under the assumption that genetic aging effects are shared between the sexes, we would expect to see similar associations with the GRS for ANM in both sexes. Female specific effects would suggest either the ANM DDR variants are specifically related to women and their reproductive capacity or that the results reflect sex-specific changes in behaviours or mental health associated with experiencing menopause at a particular age.

The statistical analyses were conducted using Stata version 15 (Statacorp, Texas) and R version 3.5.1 (R Foundation, www.R-project.org). All analytical code is available in Online resource 1.

## Results

The mean ANM was 50 years (standard deviation 5 years). The GRS including all 267 SNPs explained 7.3% of the variation in ANM (F-statistic 8126) (Supplementary Table 1). The GRS including DDR SNPs explained 4.4% of the variation, while the GRS including non-DDR SNPs explained 3.6% of the variation (Supplementary Table 1). The coefficients for the effect of each SNP on ANM is shown in Supplementary Table 2. The GRS for ANM was not associated with study centre or genotyping chip after adjusting for the first ten genetic principal components (*P *value 0.96 for chip and 0.3 for study centre). There was a modest correlation between the GRS for ANM and age at recruitment (r = − 0.01; *P *value 0.004).

### MR-pheWAS

**Fig. 2:**
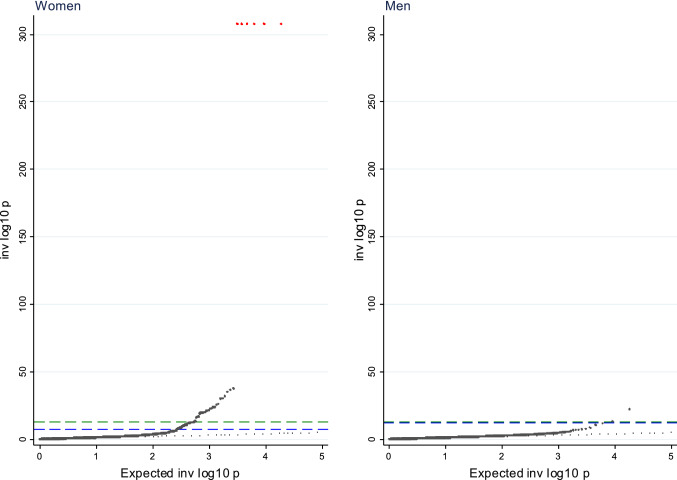
QQPLOT MR-pheWAS of age at natural menopause genetic risk score among men and women. The green line reflects the Bonferroni threshold (women 2.64 × 10^–6^; men 2.75 × 10^–6^), while the blue line reflects the false discovery rate 5% threshold (women 5.83 × 10^–4^; men 5.49 × 10^–6^)

**Fig. 3:**
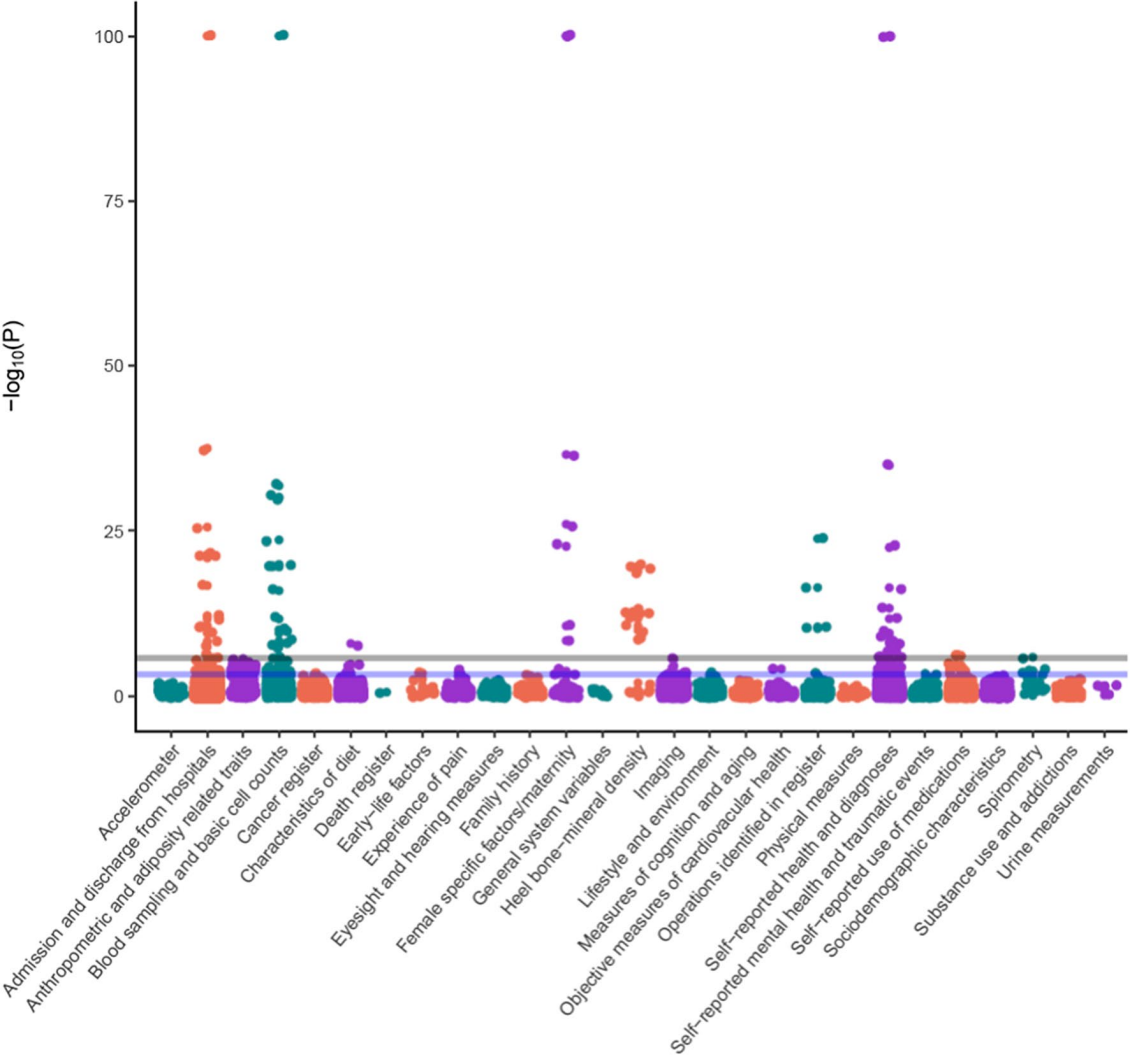
MR-pheWAS plot showing the overall distribution of findings according to categories. The grey line reflects the Bonferroni threshold (2.64 × 10^–6^), while the blue line reflects the false discovery rate 5% threshold (5.83 × 10^–4^)

Of the 18,961 tests performed, our MR-pheWAS analysis identified potential effects of ANM on 221 traits (1.17% of all traits) at a 5% false discovery rate (*P *value ≤ 5.83 × 10^–4^), and 91 (0.48%) potential effects when using the more stringent Bonferroni threshold (*P *value ≤ 2.64 × 10^–6^). A quantile–quantile plot of *P *values is shown in Fig. 2. This shows notable deviation at very significant *P *values, indicating several potential pheWAS associations. The distribution of findings across categories of traits is shown in Fig. 3, while a detailed list describing the findings available in Supplementary Table 3. Our findings included 19 traits seemingly directly related to ANM, such as starting hormone-replacement therapy, age at hysterectomy, oophorectomy, oeastradiol levels and SHBG levels. Other findings included measures related to gynaecological conditions such as endometriosis [36], general menstrual cycle characteristics (7 traits), liver function (3 traits), kidney function (4 traits), bone-mineral density and osteoporosis (66 traits), lung function (4 traits), gastrointestinal conditions (4 traits), cardiometabolic health (23 traits), breast cancer (6 traits), blood-cell composition (19 traits) and unclear/unspecified (30 traits). When restricting the GRS for ANM to the 151 strictly unrelated SNPs, we observed 147 associations at a 5% false discovery rate, out of which 51 reach a strict Bonferroni correction (Supplementary Table 4). All findings in this sensitivity analysis were among the findings in the main analysis.

**Fig. 4:**
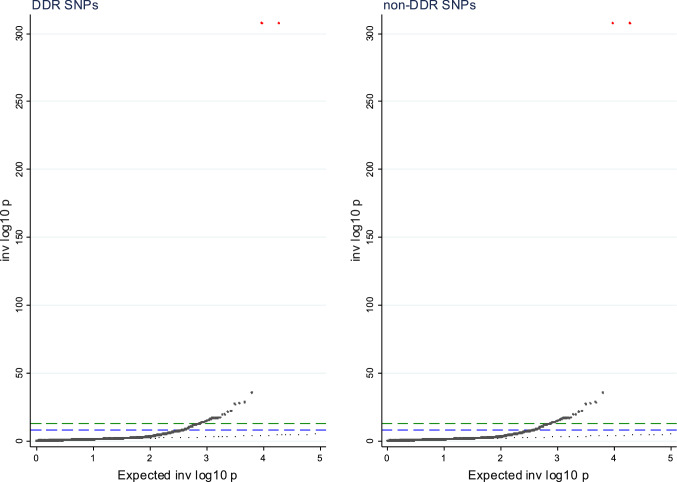
QQPLOT MR-phewas of age at natural menopause genetic risk score among women stratified by DDR and non-DDR SNPs. The green line reflects the Bonferroni threshold (2.64 × 10^–6^), while the blue line reflects the false discovery rate 5% threshold (DDR 4.54 × 10^–4^; non-DDR 2.69 × 10^–4^)

When we restricted the GRS to DDR SNPs, we observed 172 findings that were significant at the FDR 5%, out of which 91 were significant at the more stringent Bonferroni correction level (Supplementary Table 5). In contrast, when we estimated GRS to non-DDR SNPs, there were only 102 findings at the FDR 5% level and 48 at the Bonferroni level (Supplementary Table 6). Figure 4 shows the QQ-plot for the analyses of the GRS including the DDR and non-DDR findings, respectively. The full results are presented in Supplementary Tables 5 (DDR) and 6 (non-DDR). Overall, the findings from the analysis of the GRS including DDR SNPs was very similar to the main analyses including all available SNPs. When we looked at the 221 findings at the FDR 5% level from the main analysis, the coefficients tended to be fairly similar when for the two GRS, but the precision was poorer for the GRS only including non-DDR SNPs (Supplementary Table 7) (Fig. 4).

### Follow-up and replication analyses of MR-pheWAS findings

**Fig. 5:**
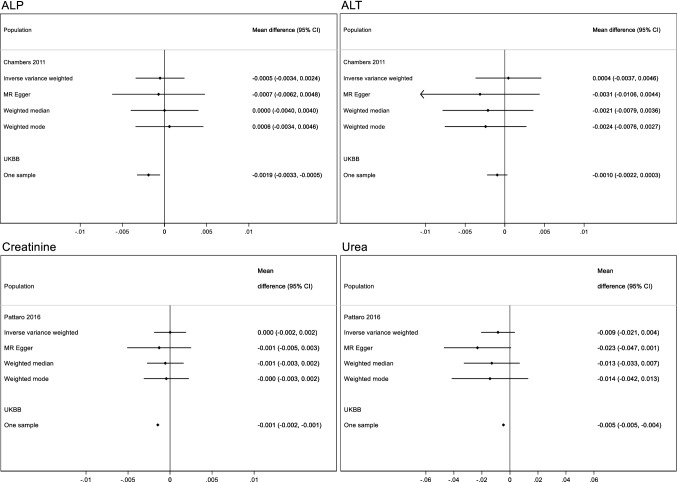
One/two-sample replication analysis of associations of age at natural menopause with kidney and liver function tests. Effect estimates reflect the differences in the outcomes per year decrease in age at menopause. The mean difference in alkaline phosphatase (ALP) and alanine transaminase (ALT) are naturally log transformed measured in *U*/*L* and adjusted for age and genomic principal components. The mean difference in creatinine and urea are expressed as differences in residuals of the log transformed measures in umol/L adjusted for age and genomic principal components. The adjustment strategy reflect that which was used in the published GWAS study of the outcomes of interest

**Fig. 6:**
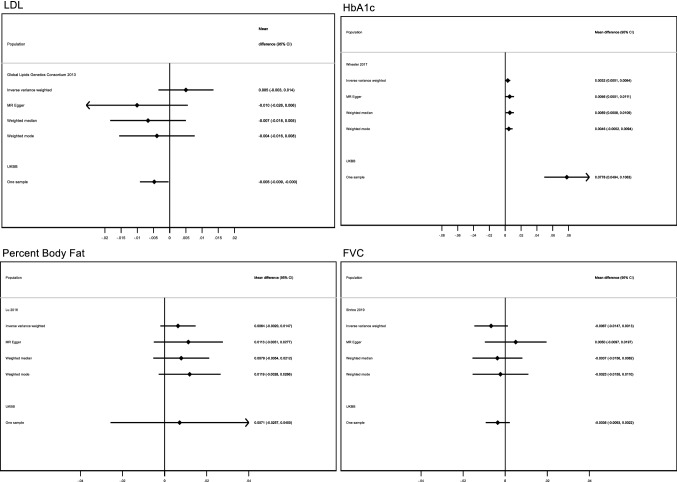
One/two-sample replication analysis of associations of age at natural menopause with LDL-cholesterol, HbA1c, percent body fat and FVC. All effects indicate the association with a one year decrease in age at menopause. The effect estimate for LDL cholesterol reflects the mean difference in the residuals for the measure in mmol/L while HbA1c was measured in mmol/mol. All estimates were adjusted for age, age squared and genomic principal components. FVC was standardized by age, height, smoking status, and genomic principal components. The adjustment strategy reflect that which was used in the published GWAS study of the outcomes of interest

**Fig. 7:**
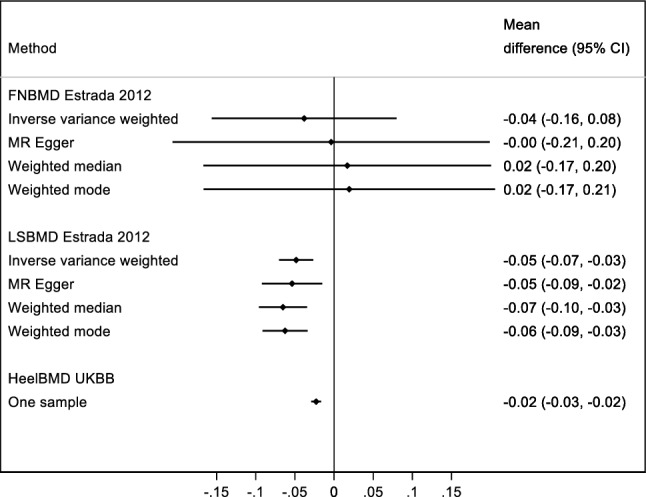
One/two-sample replication analysis of associations of age at natural menopause with femoral neck (FNBMD), lumbar spine (LSBMD) and heel (HeelBMD) bone-mineral density. All effect estimates reflect the difference in the standard deviation scores for bone-mineral density per year decrease in age at menopause adjusted for age, BMI and genomic prindipal components. The adjustment strategy reflect that which was used in the published GWAS study of the outcomes of interest

**Fig. 8:**
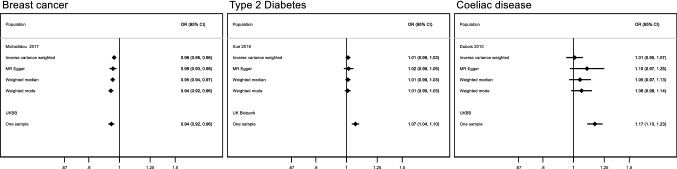
One/two-sample replication analysis of associations of age at natural menopause with breast cancer, type 2 diabetes and coeliac disease. All effect estimates reflect the odds ratio (OR) of the condition of interest per year increase in age at natural menopause. The estimates are adjusted for age and genomic principal components. The adjustment strategy reflect that which was used in the published GWAS study of the outcomes of interest

Overall, the estimates from the two-sample MR yielded effect estimates in a similar direction for the relationships between younger ANM and alkaline phosphatase (ALP), alanine transaminase (ALT), creatinine, urea, percent body fat, FVC and LDL cholesterol (Figs. 5 and 6), although the estimates were imprecise and included the null value. We did replicate our findings that younger ANM is associated with higher levels of HbA1c (adjusted mean difference 0.003 mmol/mol; 95% CI 0.0001, 0.006 per year decrease in ANM), lower odds of breast cancer (adjusted OR 0.96; 95% CI 0.95, 0.98 per year decrease in ANM), and lower levels of bone-mineral density (adjusted mean difference − 0.05; 95% CI − 0.07, − 0.03 per year decrease in ANM for lumbar spine bone-mineral density) (Figs. 6, 7 and 8). These findings were robust to the sensitivity analyses conducted in the two-sample MR. The replication analyses yielded no robust evidence of a relationship between ANM and type 2 diabetes or coeliac disease (Fig. 8).

### Negative control analysis

The negative control analysis included 155,709 men. The QQ-plot for the analysis of the GRS for ANM in men is presented in Fig. 2 and unlike the equivalent plot for women shows no substantial deviation from expected were all associations null. We found 30 findings at the FDR 5% level, out of which 11 also reached Bonferroni significance level. The findings in men included HbA1c, insulin dependent diabetes, cholesterol, triglycerides and coeliac disease. The full results for men are presented in Supplementary Table 8.

## Discussion

In our MR-pheWAS of ANM in UK Biobank, we found that a genetic predisposition to younger ANM was associated with a variety of menopause and menstrual related characteristics, in addition to lower bone-mineral density, higher risk of breast cancer, poorer kidney and liver function, differences in blood cell composition, measures of cardiometabolic health (such as lower LDL cholesterol and higher HbA1c), gastrointestinal conditions, and lung function, in addition to other traits (such as both eyes being present, leg pain on walking and never eating wheat products). We were able to replicate our findings for bone-mineral density, breast cancer and HbA1c in independent cohorts. For the traits we did not clearly replicate, estimates were directionally consistent with the MR-pheWAS results but imprecise, and it is possible that larger samples with GWAS data are needed. The only exception was LDL-cholesterol which yielded estimates in opposing directions in UK Biobank and the replication cohort. We only observed a few associations with the GRS for ANM among men, with the QQ-plot being notably different to that observed in women.

A relationship between younger ANM and lower bone-mineral density is well established from traditional observational studies [2, 3]. Due to the importance of female reproductive hormones in the maintenance of bones, bone loss sharply accelerates during the late peri-menopausal period [33]. Despite the clear association between the menopausal transition and bone-mineral density, the exact relationship with fracture risk is less clear, with studies showing inconsistent findings [34–37]. The recent GWAS of ANM also highlighted the relationship between the genetic risk for earlier ANM and a lower total bone-mineral density [19]. However, they did not replicate this estimate in an independent cohort. We therefore provide further evidence that earlier ANM appears to have a causal relationship with lower bone-mineral density in the lumbar spine, which is also a marker of osteoporosis and risk of fractures [38].

Our findings also suggest that the association between ANM and breast cancer might be causal [4]. Interestingly, this meta-analysis reported a 5% increased risk of breast cancer per year increase in ANM which is very similar to what we observed in both one-sample MR in UK Biobank and the two-sample MR in the independent replication study. The relationship between the GRS for ANM and risk of breast cancer in UK Biobank was also highlighted in the recent GWAS of ANM [19]. There is evidence that oestrogen-receptor positive and lobular cancers might be more sensitive to female sex hormones than oestrogen receptor-negative and ductal cancers. For example, the meta-analysis found that ANM was more strongly related to oestrogen-receptor positive and lobular breast tumors [4], and post-menopausal women who use hormone-replacement therapy have a greater risk of oestrogen-receptor positive than oestrogen-receptor negative tumors [39, 40].

Our finding that younger ANM is associated with higher levels of HbA1c is in line with existing evidence from studies suggesting a higher risk of type 2 diabetes and poorer glycemic control among women with a younger ANM [41, 42]. Sparse evidence also exists indicating that HbA1c levels increase after menopause [43, 44]. These associations might be explained by an influence of female sex hormones on pancreatic β-cell function, which play a major role in glucose metabolism and consequently the risk of diabetes [45]. Evidence also supports the notion that hormone-replacement therapy among peri or post-menopausal women lowered the risk of developing new-onset type 2 diabetes after menopause [46]. Our replication analysis indicated no strong evidence of a relationship between ANM and risk of type 2 diabetes, although this could reflect limited statistical power.

We were not able to follow-up all of the potentially relevant findings, either because GWAS for the traits do not currently exist, are only available in UK Biobank, or we were not able to access full summary data (e.g. blood cell composition). Several other health related traits were related to the GRS for ANM in our MR-pheWAS, and their causal relationship with ANM can be further explored in future studies. This includes for example the blood cell composition measures, calcium levels, measures of cardiometabolic health and albumin levels.

In common with most existing MR studies, we did not explore potential nonlinear effects of ANM on outcomes. The reason we have not done this and it is rarely done in other studies is because current methods are only feasible in one sample MR and require very large sample sizes, and the choice of where to put thresholds (for examining MR effects in subsets across the distribution) is unclear [47]. As large biobanks make their data available and methods are further developed, this could be further explored in future studies that follow up specific findings from our MR-pheWAS.

One limitation of our analysis is the low response in UK Biobank (5.5%), which could have resulted in selection bias [48, 49]. Participants in UK Biobank have been shown to be healthier and of a higher socioeconomic position compared to other estimates for the British population. This could have resulted in a lower burden of some of the health-related outcomes evaluated, such as a lower proportion of smokers, lower mean BMI, lower overall CVD risk (less diabetes and less hypertension), and less psychological problems, among others. The effect of potential selection bias likely varies across the large number of health-related outcomes evaluated. Reassuringly, the mean ANM was as expected based on the estimated ANM in the general population (~ 50 years) [50]. We relied on publicly available summary statistics for the replication analyses, and sex-specific results from the outcome GWAS were used where they were available. If there were sex differences in the SNP-outcome associations that were not detected and reported in the published GWAS’, this could have biased our results, most likely towards the null. Other limitations of our analyses includes the lower statistical power for some of our replication analyses, lack of generalizability to other ancestry groups than Europeans, in addition to the fact that we had to rely on sex-combined results for the majority of the replication analyses. With regard to generalizability, the relationships we present here are only generalizable to women of European ancestry, and should therefore be examined in other ancestry groups in future studies.

Our results suggest that younger ANM has potential effects on a broad range of health-related traits. Follow-up analysis indicated evidence of an effect of younger ANM on bone-mineral density, HbA1c and the risk of breast cancer. Future studies are needed to investigate the potential effects which we could not follow-up here. Additional studies using other designs with different biases and sufficient statistical power to replicate our findings would also be useful. Where future studies provide strong evidence for causal effects of ANM on several outcomes, studies to explore potential modifiable mechanisms to mitigate any effect of ANM will be important.

## Supplementary Information

Below is the link to the electronic supplementary material.

Supplementary file1 (DOCX 11 kb)

Supplementary file2 (7Z 7 kb)

Supplementary file3 (XLSX 9949 kb)

## Data Availability

Study data are available on application to UK Biobank (https://www.ukbiobank.ac.uk/enable-your-research/apply-for-access). All code for the statistical analyses is available from the corresponding author.
