# Supplementary material for: Identifying potential causal effects of age at menopause: a Mendelian randomization phenome-wide association study
Source: Eur J Epidemiol. 2022 Sep 3;37(9):971–82. doi: 10.1007/s10654-022-00903-3 (PMC9529691; doi:10.1007/s10654-022-00903-3)
Supplement: Supplementary file 1 — Supplementary file1 (DOCX 11 kb) [file 10654_2022_903_MOESM1_ESM.docx]

Strategy for literature search

We conducted a literature search to evaluate the number of studies conducted on the relationship between age at natural menopause and the risk of cardiovascular disease, breast cancer and depression. We conducted separate searches for each of the outcomes in turn. The search was conducted in pubmed. We used the following search terms: “later menopause” OR “age at menopause” OR “earlier menopause” OR “premature menopause” in addition to the MeSH terms cardiovascular disease, breast cancer and depression. Using these terms, we found 307 studies of age at menopause and cardiovascular disease, 517 on the relationship between age at menopause and risk of breast cancer, and only 49 studies of depression.
